# TBL1XR1 Is Highly Expressed in Gastric Cancer and Predicts Poor Prognosis

**DOI:** 10.1155/2016/2436518

**Published:** 2016-09-08

**Authors:** Fang Liu, Yuan He, Qinghua Cao, Ni Liu, Wenhui Zhang

**Affiliations:** ^1^Department of Oncology, Nanfang Hospital, Southern Medical University, Guangzhou 510515, China; ^2^State Key Laboratory of Oncology in South China, Collaborative Innovation Center for Cancer Medicine and Department of Molecular Diagnostics, Sun Yat-sen University Cancer Center, Guangzhou 510060, China; ^3^Department of Pathology, The First Affiliated Hospital of Sun Yat-sen University, Guangzhou 510080, China

## Abstract

*Objective*. To investigate the expression of transducin- (*β*-) like 1 X-linked receptor 1 (TBL1XR1) in human gastric cancer (GC) and its correlation with prognostic and biologic significance.* Methods*. TBL1XR1 mRNA expression was analyzed in gastric cancer using a microarray dataset (GSE2701) from the Gene Expression Omnibus (GEO). Immunohistochemistry (IHC) analysis of TBL1XR1 was performed on GC tissue microarray (TMA) to assess its prognostic and biological significance in 334 patients of GC.* Results*. Analysis of GSE2701 showed that the mRNA levels of TBL1XR1 were significantly elevated in primary gastric tumor and lymph node tissues than normal gastric tissues (*P* < 0.05). The same results of TBL1XR1 protein level were observed by IHC staining in 334 GC tissues. 204 of 334 (60.1%) primary gastric cancer tissues showed high expression of TBL1XR1 protein. TBL1XR1 overexpression was significantly correlated with lymph node metastasis (*P* = 0.000) and advanced TNM stage (*P* = 0.001). Moreover, high levels of TBL1XR1 predicted worse overall survival (*P* = 0.015). Multivariate Cox regression analysis indicated that high expression of TBL1XR1 was an independent prognostic factor for poor overall survival (HR, 0.525; 95% confidence interval, 0.367–0.752; *P* = 0.005).* Conclusion*. This present study demonstrates that TBL1XR1 is overexpressed in gastric cancer and may be a potential predictor and therapeutic target for GC patients.

## 1. Introduction

Gastric cancer (GC) is the leading cause of cancer-related mortality worldwide, and in general, incidence rates are highest in Eastern Asia particularly in China [1]. Despite advances in efficient surgical techniques and novel chemotherapeutic interventions, the long-term survival rate of GC patients remains poor [2]. The major reason of poor prognosis in GC patients is mostly attributed to local invasion and distal metastasis by complex molecular mechanisms [3]. In addition, although prognostic biomarkers like HER2 [4], VEGFR-2 [5], and EGFR [6] are useful in some specific population of GC patients, it is not enough to fully predict individual GC patients outcomes. Therefore, it is urgent to exploit novel molecular markers.

Transducin- (*β*-) like 1 X-linked receptor 1 (TBL1XR1), a core component of nuclear receptor corepressor (NCoR) complex, shows both corepressor and coactivator on nuclear receptor [7, 8]. It is thought to play a vital role in key cellular functions, including growth, antiapoptosis, and inflammation through mediating the exchange of corepressors [8, 9]. Recently, TBL1XR1 is reported to be overexpressed in multiple malignancies and contributes to carcinogenesis and tumor progression, including esophageal squamous cell cancer, cervical cancer, breast cancer, nasopharyngeal cancer, and hepatocellular carcinoma [10–14]. However, little is known about its role in gastric cancer. More interestingly, Liu et al. [10] reported that silencing TBL1XR1 in GC cells resulted in a significant decrease in VEGF-C expression, which is closely related with GC metastasis and poor prognosis [15, 16]. All of this information aroused us to address the clinical significance and potential role of TBL1XR1 in human GC patients.

In this study, we detected TBL1XR1 expression and characterized its clinicopathological function in a large cohort of GC tissues. We found that TBL1XR1 was overexpressed in GC tissues in both mRNA and protein level and correlated with present lymph node metastasis and advanced TNM stage. Importantly, TBL1XR1 overexpression predicted an inferior overall survival and could be a potential independent prognostic biomarker for GC.

## 2. Materials and Methods

### 2.1. Gene Expression Profile Data of GSE2701

Gene expression profile data GSE2701 was downloaded from the open Gene Expression Omnibus database (http://www.ncbi.nlm.nih.gov/geo/) and analyzed by the Qlucore Omics Explorer (QOE 3.1) bioinformatics software (http://www.qlucore.com/). The QOE offers state-of-the-art mathematical and statistical methods, and its main features are the ease of use and speed with which datasets can be analyzed and explored [17]. The mRNA levels of TBL1XR1 from 2 cohorts of GSE2701 were explored, respectively, in primary gastric tumor, lymph node metastases, and normal tissues.

### 2.2. Patients and Tissue Specimens

For immunohistochemistry study, paraffin-embedded pathological specimens from 334 patients with gastric cancer were obtained from the archives of the First Affiliated Hospital of Sun Yat-sen University, Guangzhou, China, between 2001 and 2006. The cases were selected based on three criteria: (i) a distinctive pathological diagnosis of gastric adenocarcinoma, (ii) primary and curative resection of the tumor without preoperative anticancer treatment, and (iii) the availability of resection tissue and follow-up data. Postsurgical chemotherapies were performed depending on the severity of the disease and according to the National Comprehensive Cancer Network (NCCN) guidelines. Clinicopathological characteristics for these patients, including gender, age at surgery, tumor size, histological type, TNM stage, and lymph node metastasis status, are detailed in Table 1. All the samples were collected with patient's informed consent after approval from the Institute Research Medical Ethics Committee of the First Affiliated Hospital, Sun Yat-sen University.

### 2.3. Tissue Microarray Construction

Tissue microarrays (TMAs) were constructed according to the method described previously [18]. Briefly, a hollow needle was utilized to punch and remove bipartite cylinders tissue core (1.0 mm in diameter) from selected donor tissue regions. Further, the punched tissue cores were inserted into a recipient paraffin block with a precisely spaced array pattern, using an automatic tissue arraying instrument (Beecher Instruments, Silver Spring, Maryland, USA). For each sample, two cores from the selected tumor area and one core from adjacent nontumor mucosa were used to construct the TMA.

### 2.4. Immunohistochemical Staining

Immunohistochemical staining was performed on formalin fixed paraffin-embedded tissues using a standard streptavidin-biotin-peroxidase complex method as previously described [18]. TMA slides were incubated at 4°C in a moist chamber overnight with rabbit polyclonal antibody against human TBL1XR1 (1 : 100, Abcam, ab190796). Staining with PBS instead of primary antibody against TBL1XR1 was used as negative control.

Nuclear immunoreactivity for TBL1XR1 protein was classified as positive signal. The scores were determined by combining the proportion of positively stained tumor cells and the intensity of staining as previously described [10]. The proportion of positively stained tumor cells was scored as follows: no positive tumor cells (score 0); <10% positive tumor cells (score 1); 10–35% positive tumor cells (score 2); 35–75% positive tumor cells (score 3); >75% positive tumor cells (score 4). Staining intensity was graded according to the following standard: no staining (score 1); weak staining (score 2); moderate staining (score 3); strong staining (score 4). The staining index was calculated as the staining intensity score multiplied by the proportion of positively stained tumor cells (values from 0 to 16). The staining was assessed by two independent pathologists (Qinghua Cao and Ni Liu) without knowing the identity of the samples. If there was a discrepancy in individual evaluations, then the two pathologists reevaluated the slides together to reach a consensus.

### 2.5. Statistical Analysis

Statistical analysis was performed with the SPSS package (version 20.0). The associations between TBL1XR1 expression and clinicopathological features were analyzed by chi-square (*χ* ^2^) test. Kaplan-Meier analysis was used for univariate survival analysis and log-rank test was applied to compare different survival curves. The multivariate Cox regression model was used to assess the potential independent prognostic factors and 95% confidence intervals (CI) of hazard ratio (HR). Statistical significance was initially set at *P* < 0.05.

## 3. Results

### 3.1. Analysis of the mRNA Level of TBL1XR1 from GEO Dataset

**Figure 1:**
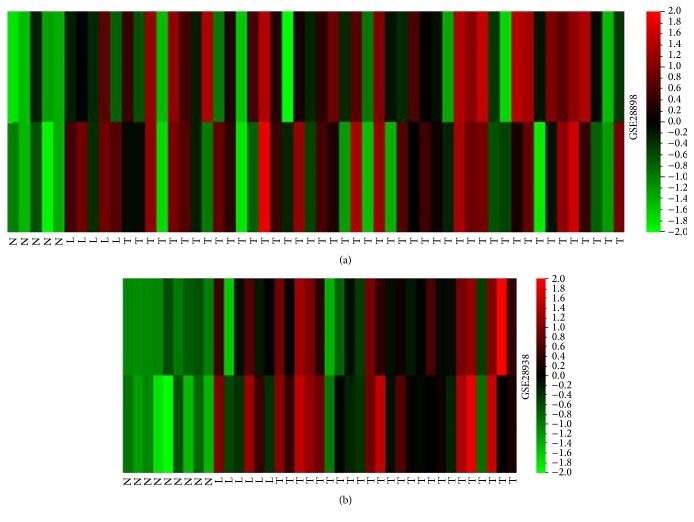
Analysis of the mRNA level of TBL1XR1 from GSE2701 dataset showed that TBL1XR1 was significantly higher in primary gastric cancer (GC) tumor (T) and lymph node metastasis lesions (L) than in normal gastric tissues (N) (*P* < 0.05). (a) Cohort 1: T + L versus N: *P* < 0.05; T versus L: *P* > 0.05. (b) Cohort 2: T + L versus N: *P* < 0.05; T versus L: *P* > 0.05.

GSE2701 consisted of 126 gastric tissues including 90 primary gastric cancers, 14 lymph node metastasis lesions, and 22 normal gastric mucosae. To compare the expression of TBL1XR1 mRNA in human gastric cancer tissues and nontumor mucosa, heat maps were made by QOE 3.1 showing the detail of TBL1XR1 mRNA expression. As shown in Figure 1, TBL1XR1 was significantly higher in primary GC and lymph node metastasis lesions than normal gastric tissues, respectively (*P* < 0.05). In addition, there was no significant expression difference between primary gastric tumor and lymph node samples (*P* > 0.05).

### 3.2. The Expression of TBL1XR1 Protein in Gastric Cancer and Adjacent Nontumor Mucosa

**Figure 2:**
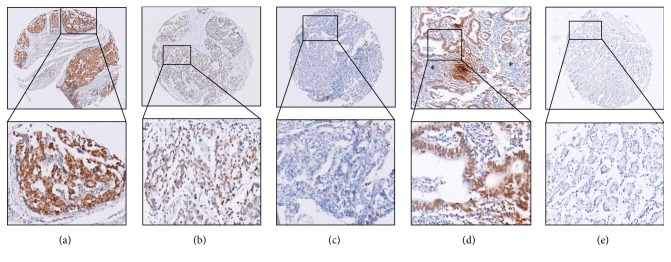
Expression of TBL1XR1 protein in GC tissue, lymph node metastasis lesions, and adjacent nontumor mucosal tissues. Immunohistochemistry staining revealed TBL1XR1 high expression (a, b) and low expression (c) in GC tissues, high expression in lymph node metastasis lesion (d) (*∗*: lymphoid tissue around metastatic cancer), and low expression in adjacent nontumor mucosal tissues (e) ((a) scoring index = 16; (b) scoring index = 12; (c) scoring index = 4; (d) scoring index = 16; (e) scoring index = 0).

In order to examine whether TBL1XR1 protein was overexpressed in gastric cancer, immunohistochemistry staining was performed in 334 GC tissues, 30 corresponding lymph node metastasis lesions, and 20 adjacent nontumor mucosa tissues. Samples with staining index ≥8 (median score of TBL1XR1 expression in the gastric cancers) were determined as high expression and samples with staining index <8 were determined as low expression. High expression of TBL1XR1 protein was detected in 204 of 334 (60.1%) primary gastric cancer tissues (Figures 2(a) and 2(b)) and 19 of 30 (63.3%) lymph node metastasis lesions (Figure 2(d)) while 18 of 20 (90.0%) adjacent nontumor mucosae showed only low expression (Figure 2(e)) (*P* < 0.001, *P* < 0.001, resp.).

### 3.3. Association of TBL1XR1 Protein Expression with Clinicopathological Parameters

The high or low expression rates of TBL1XR1 protein in gastric cancer with respect to several standard clinicopathological features are presented in Table 1. It was showed that TBL1XR1 protein overexpression was significantly related with advanced TNM stage (*P* = 0.001) and present lymph node metastasis (*P* = 0.000). There was no significant correlation between TBL1XR1 protein expression and the other clinicopathological parameters, such as gender, age at surgery, tumor size, and histological type (*P* > 0.05) (Table 1).

### 3.4. The Relationship between TBL1XR1 Protein Expression, Clinicopathological Features, and Overall Survival: Univariate Survival Analysis

**Figure 3:**
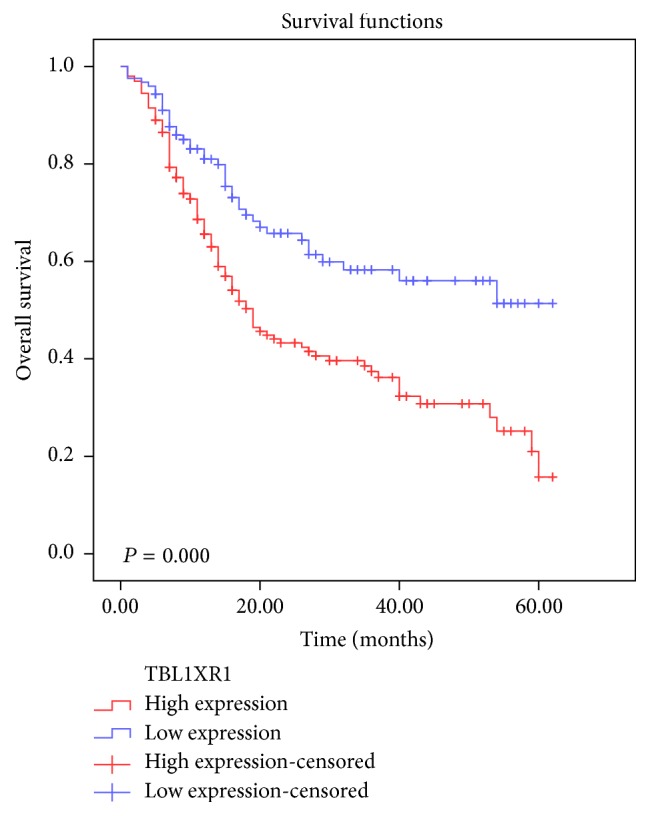
Survival curve for 334 gastric cancer patients according to TBL1XR1 protein expression status (log-rank test). High expression of TBL1XR1 protein was closely correlated with inferior overall survival (OS) (*P* = 0.000).

To confirm the representativeness of the gastric cancers in our study, we analyzed established prognostic factors of patient survival. The Kaplan-Meier analysis demonstrated that there was a significant impact of the well-known clinicopathological prognostic parameters, such as histological type (*P* = 0.006), TNM stage (*P* = 0.000), and lymph node metastasis status (*P* = 0.003) on patients' overall survival (Table 2). Moreover, overall survival was significantly impaired in patients with high expression of TBL1XR1 protein compared to patients with low expression of TBL1XR1 protein in tumors (*P* = 0.000) (Table 2, Figure 3). In this regard, the mean value of overall survival time was 41.53 months in patients with low expression of TBL1XR1 compared to 28.99 months in patients with high levels of TBL1XR1 (Table 2).

### 3.5. Independent Prognostic Factors of Gastric Cancer Patients: Multivariate Cox Regression Analysis

A multivariate regression analysis based on the Cox proportional hazard model was used to test the independent value of each parameter in predicting overall survival. The expression of TBL1XR1 protein and other clinicopathological features that were significant by univariate analysis (histological type, TNM stage, and lymph node metastasis status) were included in the multivariate analysis. The results showed that high expression of TBL1XR1 was an independent prognostic factor for poor overall survival (hazard ratio, 0.525; 95% confidence interval, 0.367–0.752; *P* = 0.005), as well as histological type, TNM stage, and lymph node metastasis status (*P* = 0.048, *P* = 0.000, and *P* = 0.001, resp.) (Table 2).

## 4. Discussion

Although TBL1XR1 is thought to be involved in carcinogenesis and tumor progression in multiple studies, the relationship between TBL1XR1 and GC is unclear. In the present study, we demonstrated the oncological significance of TBL1XR1 in GC patients: (a) the expression of TBL1XR1 was significantly elevated in primary gastric tumor and lymph node tissues; (b) high levels of TBL1XR1 were associated with lymph node metastasis and advanced TNM stage; (c) TBL1XR1 overexpression is a negative predictor of prognosis in GC patients.

TBL1XR1, a transcriptional cofactor, plays an important role in controlling precise switch between gene repression and activation in transcriptional regulation [10, 19]. Consistent with our study, it was reported that TBL1XR1 was elevated in lung squamous cell cancer, esophageal squamous cell cancer, cervical cancer, breast cancer, nasopharyngeal cancer, and hepatocellular carcinoma [10–14, 20], suggesting that TBL1XR1 upregulation might be a common event in the progression of different cancers. In addition, we demonstrated that TBL1XR1 protein expression was significantly related with lymph node metastasis, advanced tumor stage, and poor prognosis but was not with gender, age at surgery, tumor size, and histological type. These findings were almost in line with previous studies. However, the study from Kuang et al. [14] indicated that TBL1XR1 expression was associated with clinical stage, prognosis, tumor size, and histological grade. Li et al. [12] showed that TBL1XR1 expression was markedly related with pathological differentiation and cerbB2 expression besides tumor stage and prognosis. These results indicated that, even sharing the similar prognosis prediction, the biological behavior and anatomical characteristics of TBL1XR1 might be distinguished among the tumor subtypes.

Moreover, accumulated studies suggest that TBL1XR1 may play an important role in tumorigenesis, invasion, metastasis, and developing resistance to therapies and has potential to be a negative prognosis biomarker in various cancers. Li et al. [12] reported that TBL1XR1 overexpression promoted whereas TBL1XR1 silencing inhibited proliferation and tumorigenicity in breast cancer cells in vitro and in vivo. Liu et al. [10] found that TBL1XR1 overexpression provoked lymphangiogenesis and lymphatic metastasis in ESCC and may represent a novel prognostic biomarker and therapeutic target. In nasopharyngeal cancer, Chen et al. [13] showed that increased TBL1XR1 expression suppressed cisplatin-induced apoptosis and may serve as an effective biomarker for selective therapeutic regimen for NPC patients. However, in contrast to these studies, Daniels et al. [19] demonstrated that TBL1XR1 was downregulated in prostate cancer and inhibited prostate cancer growth as a coactivator of androgen receptor. Also, TBL1XR1 downregulation was found in acute lymphoblastic leukemia with positive ETV6-RUNX1 fusion [21]. This discrepancy of TBL1XR1 expression may relate with cancer type, TBL1XR1 gene amplification, or focal deletions [22], suggesting the complex role of TBL1XR1 in cancer progression.

Considerable researches have been carried out to determine the role and mechanism of TBL1XR1 in cancers. For instance, TBL1XR1 was involved in lung carcinogenesis by immortalizing a bronchial epithelial cell line that was considered “precancerous” [23]. It was reported that TBL1XR1 promoted cervical cancer cell metastasis by NF-kB and Wnt/*β*-catenin pathways to induce epithelial-to-mesenchymal transition (EMT) [11]. Furthermore, TBL1XR1 activated NF-*κ*B as to suppress cisplatin-induced apoptosis of nasopharyngeal cancer cells [13]. In ESCC cells, TBL1XR1 was demonstrated as an upstream target gene for VEGF-C and induced VEGF-C expression [10] and promoted lymphangiogenesis and lymph node metastasis. Interestingly, in GC, colorectal cancer, and breast cancer besides ESCC, analysis of datasets from GEO showed that TBL1XR1 levels were significantly correlated with VEGF-C expression, and silencing TBL1XR1 resulted in decrease in VEGF-C expression. These findings implied that TBL1XR1 contributed to lymphangiogenesis and lymph node metastasis through activating VEGF-C which may be a common mechanism for tumor metastasis in multiple cancer types, including GC. However, further studies should be performed to verify the role of TBL1XR1 in GC metastasis and related mechanisms.

In conclusion, our study showed that TBL1XR1 is overexpressed in gastric cancer in mRNA and protein level. Elevated TBL1XR1 expression is correlated with lymph node metastasis and advanced tumor stage and may serve as an independent factor for poor prognosis in GC patients.

## Figures and Tables

**Table 1:** Correlation of TBL1XR1 protein expression with clinicopathological parameters.

Variable	All cases	TBL1XR1 protein expression		*P* value^a^
		High	Low	
Gender				
Male	233	134	99	0.051
Female	101	70	31	
Age at surgery				
<57^b^	172	109	63	0.432
≥57	162	95	67	
Tumor size				
≥5 cm	149	102	47	0.013
<5 cm	185	102	83	
Histological type				
Intestinal	265	162	103	0.537
Diffuse	69	42	27	
TNM				
I + II	117	5	112	**0.001**
III + IV	217	204	13	
Lymph node metastases				
Present	234	174	60	**0.000**
Absent	100	30	70	

^a^Chi-square test; ^b^median age.

**Table 2:** Univariate and multivariate analysis of different prognostic factors for overall survival of patients with gastric cancer.

Variable	All cases	Univariate analysis		Multivariate analysis		
		Mean ± SE^a^	*P* value^b^	HR^c^	95% CI^d^	*P* value
Gender						
Male	233	35.66 ± 1.87	0.053			
Female	101	28.83 ± 2.59				
Age at surgery						
<57^e^	172	32.34 ± 2.14	0.312			
≥57	162	35.16 ± 2.20				
Tumor size						
≥5 cm	149	31.42 ± 2.21	0.289			
<5 cm	185	35.35 ± 2.11				
Histological type						
Intestinal	265	35.04 ± 1.68	**0.006**	1.507	1.004–2.262	**0.048**
Diffuse	69	24.81 ± 3.22				
TNM						
I + II	117	52.14 ± 2.15	**0.000**	1.862	1.348–2.573	**0.000**
III + IV	217	24.73 ± 1.64				
Lymph node metastases						
Present	234	25.73 ± 1.61	**0.003**	0.286	0.139–0.590	**0.001**
Absent	100	54.34 ± 1.53				
TBL1XR1 protein expression						
High	204	28.99 ± 1.86	**0.000**	0.525	0.367–0.752	**0.005**
Low	130	41.53 ± 2.48				

^a^SE, standard error; ^b^Chi-square test; ^c^HR, hazard ratio; ^d^CI, confidence interval; ^e^median age.

## References

[1] Torre L. A., Bray F., Siegel R. L., Ferlay J., Lortet-Tieulent J., Jemal A. (2015). Global cancer statistics, 2012. *CA—A Cancer Journal for Clinicians*.

[2] Bertuccio P., Chatenoud L., Levi F. (2009). Recent patterns in gastric cancer: a global overview. *International Journal of Cancer*.

[3] Hsu N.-Y., Chow K.-C., Chen W.-J., Lin C.-C., Chou F.-F., Chen C.-L. (1999). Expression of nm23 in the primary tumor and the metastatic regional lymph nodes of patients with gastric cardiac cancer. *Clinical Cancer Research*.

[4] Bang Y. J., Van Cutsem E., Feyereislova A. (2010). Trastuzumab in combination with chemotherapy versus chemotherapy alone for treatment of HER2-positive advanced gastric or gastro-oesophageal junction cancer (ToGA): a phase 3, open-label, randomised controlled trial. *The Lancet*.

[5] Fuchs C. S., Tomasek J., Yong C. J. (2014). Ramucirumab monotherapy for previously treated advanced gastric or gastro-oesophageal junction adenocarcinoma (REGARD): an international, randomised, multicentre, placebo-controlled, phase 3 trial. *The Lancet*.

[6] Satoh T., Lee K. H., Rha S. Y. (2015). Randomized phase II trial of nimotuzumab plus irinotecan versus irinotecan alone as second-line therapy for patients with advanced gastric cancer. *Gastric Cancer*.

[7] Zhang X.-M., Chang Q., Zeng L., Gu J., Brown S., Basch R. S. (2006). TBLR1 regulates the expression of nuclear hormone receptor co-repressors. *BMC Cell Biology*.

[8] Perissi V., Aggarwal A., Glass C. K., Rose D. W., Rosenfeld M. G. (2004). A corepressor/coactivator exchange complex required for transcriptional activation by nuclear receptors and other regulated transcription factors. *Cell*.

[9] Hoberg J. E., Yeung F., Mayo M. W. (2004). SMRT derepression by the I*κ*B kinase *α*: a prerequisite to NF-*κ*B transcription and survival. *Molecular Cell*.

[10] Liu L., Lin C., Liang W. (2015). TBL1XR1 promotes lymphangiogenesis and lymphatic metastasis in esophageal squamous cell carcinoma. *Gut*.

[11] Wang J., Ou J., Guo Y. (2014). TBLR1 is a novel prognostic marker and promotes epithelial-mesenchymal transition in cervical cancer. *British Journal of Cancer*.

[12] Li X., Liang W., Liu J. (2014). Transducin (*β*)-like 1 X-linked receptor 1 promotes proliferation and tumorigenicity in human breast cancer via activation of beta-catenin signaling. *Breast Cancer Research*.

[13] Chen S.-P., Yang Q., Wang C.-J. (2014). Transducin *β*-like 1 X-linked receptor 1 suppresses cisplatin sensitivity in nasopharyngeal carcinoma via activation of NF-*κ*B pathway. *Molecular Cancer*.

[14] Kuang X., Zhu J., Peng Z., Wang J., Chen Z. (2016). Transducin (beta)-like 1 X-linked receptor 1 correlates with clinical prognosis and epithelial-mesenchymal transition in hepatocellular carcinoma. *Digestive Diseases and Sciences*.

[15] Cho H. J., Kim I.-K., Park S.-M. (2014). VEGF-C mediates RhoGDI2-induced gastric cancer cell metastasis and cisplatin resistance. *International Journal of Cancer*.

[16] Cao W., Fan R., Yang W., Wu Y. (2014). VEGF-C expression is associated with the poor survival in gastric cancer tissue. *Tumor Biology*.

[17] Ji K., Ma W., Zheng W. (2014). Data mining of Gene expression profiles of Saccharomyces cerevisiae in response to mild heat stress response. *bioRxiv*.

[18] Liu F., Cao Q., Liu N. (2014). Overexpression of testes-specific protease 50 (TSP50) predicts poor prognosis in patients with gastric cancer. *Gastroenterology Research and Practice*.

[19] Daniels G., Li Y., Gellert L. L. (2014). TBLR1 as an androgen receptor (AR) coactivator selectively activates AR target genes to inhibit prostate cancer growth. *Endocrine-Related Cancer*.

[20] Liu Y., Sun W., Zhang K. (2007). Identification of genes differentially expressed in human primary lung squamous cell carcinoma. *Lung Cancer*.

[21] Parker H., An Q., Barber K. (2008). The complex genomic profile of ETV6-RUNX1 positive acute lymphoblastic leukemia highlights a recurrent deletion of TBL1XR1. *Genes Chromosomes and Cancer*.

[22] Yan H.-T., Shinka T., Kinoshita K. (2005). Molecular analysis of TBL1Y, a Y-linked homologue of TBL1X related with X-linked late-onset sensorineural deafness. *Journal of Human Genetics*.

[23] An Q., Pacyna-Gengelbach M., Schlüns K. (2003). Identification of differentially expressed genes in immortalized human bronchial epithelial cell line as a model for in vitro study of lung carcinogenesis. *International Journal of Cancer*.

